# An ethnobotanical study on medicinal plants of Shexian Dryland Stone Terraced System in northern China

**DOI:** 10.1186/s13002-022-00560-6

**Published:** 2022-10-14

**Authors:** Yu Bai, Qing Zhang, Xianlin He, Haifei Wang, Wanlin Li, Jinfeng Zhu, Yuan Meng, Chunlin Long

**Affiliations:** 1 Key Laboratory of Ecology and Environment in Minority Areas (Minzu University of China), National Ethnic Affairs Commission, Beijing, 100081 China; 2 College of Life and Environmental Sciences, Minzu University of China, Beijing, 100081 China; 3 Bureau of Agriculture and Rural Affairs of Shexian, Hebei Province Shexian, 056400 China; 4 Key Laboratory of Ethnomedicine (Minzu University of China), Ministry of Education, Beijing, 100081 China; 5 Institute of National Security Studies, Minzu University of China, Beijing, 100081 China

## Abstract

**Background:**

Shexian Dryland Stone Terraced System (SDSTS) in the Taihang Mountains was formally recognized as Globally Important Agricultural Heritage Systems (GIAHS) by Food and Agriculture Organization on May 20, 2022. People there have been relying on the terraced fields for centuries, using various plants, including medicinal plants. However, little information was reported about the flora in SDSTS, nor medicinal plants. Thus, the present study aims to identify and document medicinal plants traditionally used by the local people living around the SDSTS and associated ethnobotanical knowledge.

**Methods:**

We conducted investigations in Shexian County, Hebei Province, North China, where SDSTS is distributed. Then, Wangjinzhuang, a community located in the core zone of SDSTS, was chosen as the case site. We selected the informants through purposive and snowball sampling. The data were collected through semi-structured interviews, participant observation, and key informant interviews. The medicinal plants traditionally used by the local people were documented and analyzed. We examined and confirmed the botanical identification based on voucher specimens and by cross-checking the descriptions with the series of books, scientific papers on medicinal plants, and the plant databases.

**Results:**

The local people have rich traditional knowledge to collect and use medicinal plants in SDSTS. Records of 123 medicinal plant species belonging to 51 families were obtained from SDSTS. Asteraceae was represented by 16 species, followed by Fabaceae, Lamiaceae and Ranunculaceae. (They all have 8 species.) The majority of the reported plant species were commonly processed into decoctions. And 180 diseases affecting humans were reported to be treated with traditional medicinal plants from SDSTS.

**Conclusion:**

It is the first ethnobotanical study on medicinal plants in China-Nationally Important Agricultural Heritage Systems, and in globally important agricultural heritage systems as well. Medicinal plants are crucial for people living in Shexian County. It is necessary to recognize and respect traditional knowledge peculiar to the mountainous region of northern China, especially for those involved in the human–nature interaction and the role of knowledge in agrobiodiversity conservation and rural development that local residents have persisted for centuries.

## Introduction

Globally Important Agricultural Heritage Systems (GIAHS) are defined as “remarkable land use systems and landscapes which are rich in globally significant biological diversity evolving from the co-adaptation of a community with its environment and its needs and aspirations for sustainable development.” according to Food and Agriculture Organization of the United Nations (FAO) [1]. GIAHS have resulted not only in outstanding aesthetic beauty, maintenance of globally significant agricultural biodiversity, resilient ecosystems and valuable cultural inheritance, but sustainably provided multiple goods and services, food and livelihood security for millions of people. Thus, the focus of the GIAHS is the dynamic conservation and adaptive management of traditional agricultural systems, to protect unique and vulnerable landscapes, and to preserve traditional knowledge and cultural heritage of local farming communities [2]. By the end of May 2022, there were 65 heritages having been identified as GIAHS by FAO from 22 countries around the world. As the first country that identifies and conserves agricultural heritage systems at the national level, China ranks the top one with 18 heritages until now, starting with the Qingtian Rice-Fish Culture System in Zhejiang Province which was selected as the first GIAHS pilot site in China by FAO in 2005. With a long history of agricultural development, Chinese farmers have been performing a variety of agricultural practices suitable for different natural conditions and created splendid agricultural heritages, including agricultural landscapes, knowledge, techniques and so on [3]. The Chinese Ministry of Agriculture (MOA) initiated the designation of China-Nationally Important Agricultural Heritage Systems (China-NIAHS) in 2012, devoting to reinforcing the awareness on the values of China-NIAHS and promoting the ecological protection of heritage sites, cultural inheritance and economic development [4]. Shexian Dryland Stone Terraced System (SDSTS) had been identified as China-NIAHS in 2014. In May 2022, SDSTS was formally recognized as GIAHS by FAO, for its unique ways of using traditional practices and knowledge while maintaining local biodiversity and ecosystems.

Both GIAHS and China-NIAHS are traditional farming systems that have emerged over centuries of coevolution between indigenous farmers and their environment using inventive self-reliance, experiential knowledge, and locally available resources, which represents accumulated experiences of peasants interacting with the environment without access to external inputs, capital, or scientific knowledge [5–8]. These systems are well adapted to their particular environment with significant elements of sustainability and tend to conserve natural resource base, allowing traditional farmers to maximize harvest security under low levels of technology and with limited environmental impact based on the cultivation of various crops and varieties in time and space [9]. Therefore, these systems are of considerable importance because of the significance, the wealth and breadth of accumulated knowledge and experiences in the management and use of resources they represent. It is imperative that they be considered globally significant resources and should be protected and preserved as well as allowed to evolve under the threats of modern agriculture [10].

Rich biodiversity is one of the salient features of GIAHS or China-NIAHS. Such systems support a high degree of plant diversity in the form of polycultures and/or agroforestry patterns [5, 9]. However, diversity is maintained not only within a cultivated area, it also involves natural vegetation adjacent to their fields except for crops. Many of them are wild or weedy relatives of crop plants, from which local farmers obtain their living requirements by means of multiple usage patterns such as construction material, firewood, tools, medicines, livestock feed and human food. For example, the Hani people in the Honghe Prefecture of Southeastern Yunnan, China, have collected and used a total of 224 wild edible plants from the Hani Rice Terraced System [11]. The P’urhepecha Indians who lived around Lake Patzcuaro in Mexico have used at least 224 species of native and naturalized vascular plants for dietary, medicinal, household, and fuel needs [12]. But the most existing studies were prone to focus on peculiar cultivated species that dominated the agricultural system [13–16]. The wild plant resources growing in the surroundings are largely overlooked, which is happening to Shexian Dryland Stone Terraced System (SDSTS) as well [17].

Shexian Dryland Stone Terraced System (SDSTS) is a typical mountainous farming ecosystem that lies in the Taihang Mountains. Diverse terrains allow SDSTS to harbor abundant biodiversity including wildlife and wild herbal medicines. The vegetation regionalization of Shexian County belongs to the subregion of North China mountainous flora, located in the transition band between Taihang Mountains–Lüliang Mountains flora district and South Taihang Mountains–Zhongtiao Mountains flora district [18]. Both temperate coniferous forests and warm-temperate deciduous broad-leaved forests are existing there, with herbs and shrubs dominating the wild plants [19]. During the human and nature's coordinated development for over 700 years, a unique rain-fed agricultural system with characteristic eco-agriculture products has emerged and developed through the process of agroforestry systems. The local people intercroppingly grow millet, pulses, walnut, persimmon, Sichuan pepper (*Zanthoxylum bungeanum*), *Bupleurum* (one of the most important herbal medicinal plants in China) and other crops in the systems. There is abundant ecological intelligence of “planting crops in field, storing grain into granary, saving food from mouth.” The five-in-one compound socio-ecological system of “terraces-villagers-crops-donkeys-stone” plays a crucial ecological role in the conservation of genetic resources, biodiversity, soil, water and so on [20]. Various plants in SDSTS involving food crops, vegetable crops, and medicinal plants, serve as the primary food sources in people’s daily life and the crucial material reserve confronted with disasters and famines. People in local have gotten through a few famines due to crop failure falling back on the edible and medicinal plants in history.

As a matter of fact, Shexian Dryland Stone Terraced System (SDSTS) had received considerable attention since it was identified as China-National Important Agricultural Heritage Systems (China-NIAHS) in 2014, but seldom on medicinal plants. Studies were mainly about its farming technique [17, 20–22], soil erosion and soil element characteristics [23, 24], ecological value of landscape [19, 25, 26] and economic efficiency. Shexian dryland terraces’ origins, classifications and features were studied [22], through the systematic research to collect abundant agricultural species and traditional landraces [27], as well as the conservation and utilization experiences and associated technologies in Wangjinzhuang Community. Han focused on the diet of villagers living in the core area of SDSTS and described the whole process from planting to eating through the food system by cultivating grain on the terrace, storing grain in the house, and saving food from the mouth. The article illustrated how local residents eat the wild plants and they also collected the wild medicinal plants during famines [21]. Zhang combed the characteristics of the agricultural landscape system in Shexian County [19], indicating that the landscape system of mountain agriculture in Shexian County was comprised of nature matrix, stone terraces, settlement and culture. A rich variety of wild vegetation acted as the fundamental background, increasing the stratification of landscape and preventing flood as well.

Shexian County has been one of the most traditional Chinese medicine-cultivated counties in Hebei Province and one of the top three manufacturing locations of *Bupleurum* in China. These medicinal plants had also played a vital role in the victories of 129 Division led by Bocheng Liu and Xiaoping Deng in World War II, the Anti-Japanese war from 1937 to 1945. Among them, *Bupleurum* and *Forsythia* (another famous herbal medicine plant in China) represented remarkable contributions to healing and rescuing the wounded in the war. They exhibited a long history and a valuable tradition in making clever use of medicinal plants that appeared. As a result, medicinal plants and associated traditional knowledge composed an important proportion of the local cultural system.

Plants resources are indispensable to SDSTS, but existing researches have been keeping eye on the cultivated species, losing sight of the wild which are crucial and irreplaceable supplies when the surroundings become more tough, let alone medicinal plants. In particular, under the impulsion of modernization, more and more people prefer to head for towns. Thus, there is a devastating threat to inheriting the traditional knowledge of using medicinal plants [28]. As a representative of the mountain farming system in North China, it is significant to study and explore medicinal plants of SDSTS, and document their traditional knowledge, which may help to protect them from disappearing in a rapid-developing era.

## Methods

### Study area

**Fig. 1:**
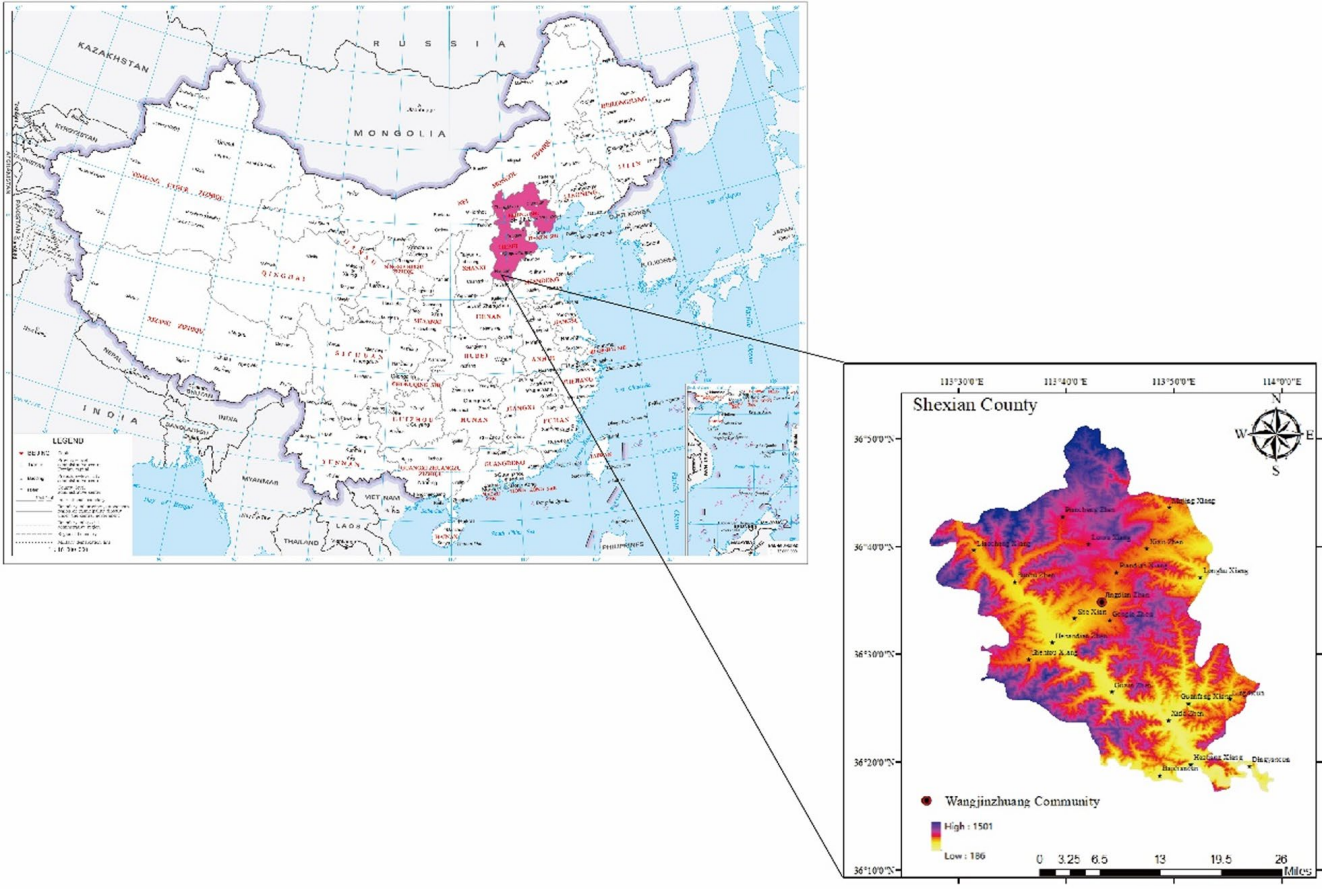
Study area

Wangjinzhuang Community, belonging to Jingdian Township, Shexian County, Hebei Province, China, is composed of 5 villages. These villages are connected with each other. It covers an area of 12 square kilometers and accommodates 4406 families [25, 26]. Wangjinzhuang has been the typical representative for SDSTS due to its wide distribution of dryland stone terraced fields and complete historical traditions. It is also the core conservation area of the heritage site located in the east of Shexian County. The terraces constructed in Wangjinzhuang Community have been called “the Second Great Wall of China” because it has a large scale of dryland stone terraces up to 8 square kilometers [20, 22]. As an agricultural heritage site, Wangjinzhuang Community has significant heritage values in addition to its splendid terraced landscape, including donkey culture, stone culture, farming culture, and revolutionary culture [29]. During the long time when local people live on the terraces in a fragile ecosystem, people still preserve and inherit the rich biodiversity-associated knowledge there, accumulating the precious, unique, varied and unsophisticated cultural resources including the traditional knowledge about medicinal plants (Fig. 1).

**Fig. 2:**
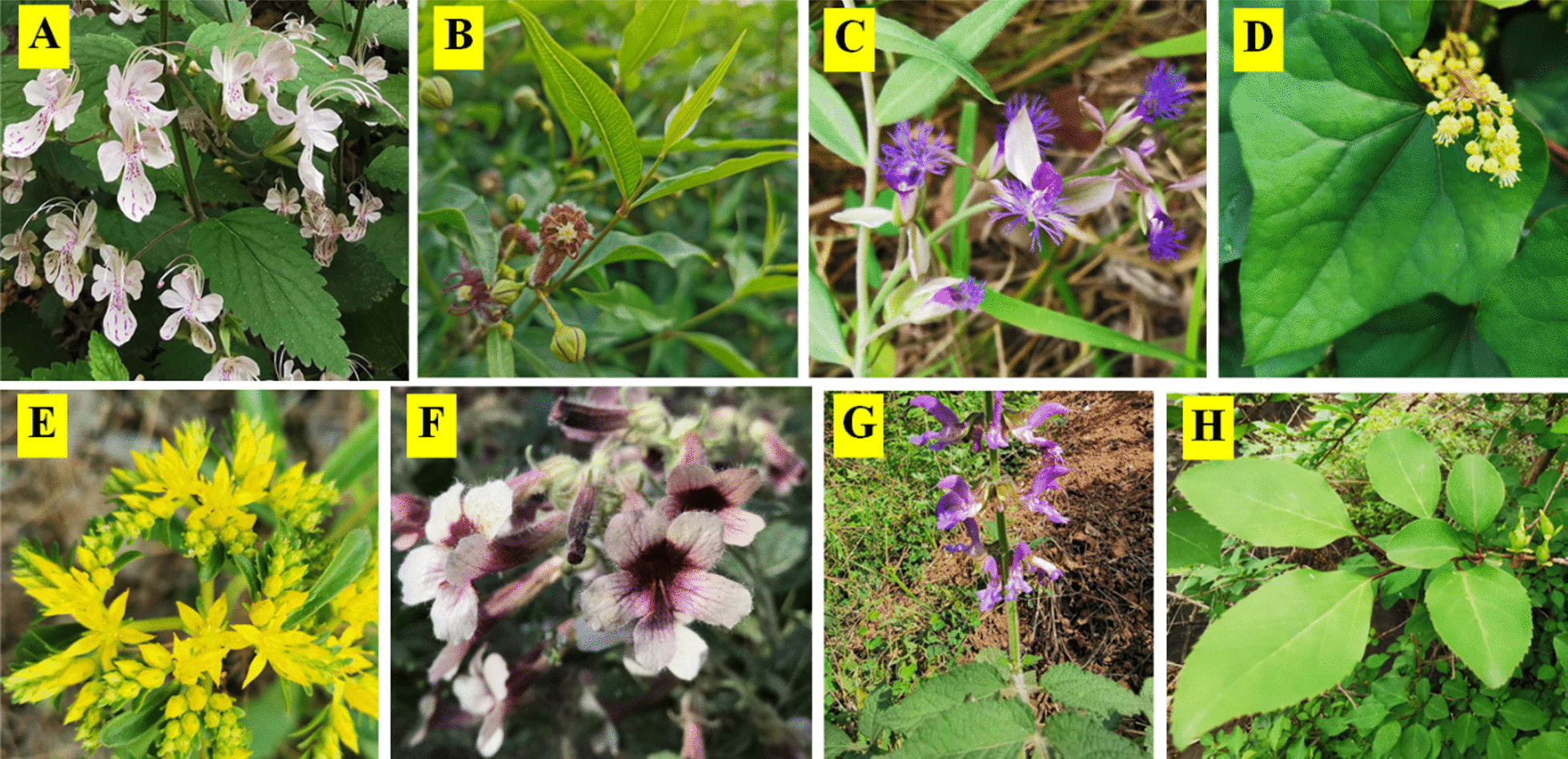
Some medicinal plants in the study area (*Schnabelia terniflora* (Maxim.) P. D. Cantino, *Periploca sepium* Bunge, *Polygala sibirica* L., *Menispermum dauricum* DC., *Phedimus aizoon* (L.)’t Hart, *Rehmannia glutinosa* (Gaert.) Libosch. ex Fisch. et Mey, *Salvia miltiorrhiza* Bunge and *Forsythia suspensa* (Thunb.) Vahl are from A to H, respectively.)

Wangjinzhuang Community lies between latitudes 36° 17′ 0″ N to 36° 55′ 0″ N, and longitudes 113° 26′ 0″ E to 114° 00′ 0″ E, which is mountainous and stony at an average elevation of 856.5 m above sea level. From the view of terrain, the community is high in the northwest and low in the southeast under a complex topographical condition, where floods and droughts are frequent due to the severe lack of soil and water on this barren land [30]. Therefore, stones can be found here and there. Wangjinzhuang Community has a northern temperate continental monsoon climate with clearly distinct dry–wet seasons, in the semiarid and semi-humid regions. The annual average temperature of the community was 13.5 °C. The coldest month is January and the hottest is July whose highest average temperature can reach 26.9 °C [26]. The annual average precipitation there is 540 mm, peculiar to SDSTS among any other terraced system as GIAHS. It provides suitable conditions for dryland crops such as *Setaria italica* (L.) Beauv. and *Glycine max* (L.) Merr., and indicates a significant interannual variation because of the topography condition, causing the rainstorm and then floods (Fig. 2).

### Informants interviewed

Five key informants were initially recruited via purposive sampling by representatives working in the local administrative authorities from the Bureau of Agriculture and Rural Affairs in Shexian County, and Conservation and Utilization Association of Shexian Dryland Stone Terraced System (CUA-SDSTS). The criterion for the sampling was being known in the community to have knowledge of medicinal plants and their use to treat ailments. Further informants were recruited thereafter by snowball sampling. The 78 informants consisted of 76 practitioners who were members in CUA-SDSTS and 2 herbalists who had their own clinics there. Informants were aged between 30 and 75, with an average age of mid-forties.

### Ethnobotanical data collection and plants identification

First, the permission for this study was supported by the Bureau of Agriculture and Rural Affairs of Shexian County and a prior informed oral consent was obtained from the informants through the administrative officials in the local government. We arranged an inventory of wild plants locally based on what the local did in the previous work before interviewing informants. Then, a verification was made by semi-structured interviews [31, 32] and field surveys. The semi-structured interviews were performed by asking informants to share information related to medicinal plants, including used plant parts, drug preparations and diseases treated [33, 34]. The frequency of use of medicinal plants was classified into “frequent,” “moderate” and “scarce” marked with “***,” “**” and “*,” respectively, and the key informants for purposes of classifying these species were selected randomly from all informants.

The field surveys were conducted between June 2020 and May 2021. We made on-the-spot investigations in Jingdian Township, Gengle Township and Guanfang Township of Shexian County, then focused on the western slope corner of Houjiao Gully for the fourth street village and Gaoyan Glover of Dishui Gully for the fifth street village after that. The herbalists firstly mentioned the medicinal plants they had used and later led us to the wild where we can find them.

Voucher specimens were collected during the field trips. They were deposited in the Herbarium of Minzu University of China. For plants identification, taxonomic nomenclature was mainly based on Plants of the World Online (https://powo.science.kew.org/) and the Plant Plus of China (http://www.iplant.cn/) databases. We also took photographs of all the medicinal plants as a special disposition for the infeasibility of the voucher collecting. All data were analyzed in Microsoft Excel.

## Results

### Diversity of medicinal plant species in Shexian Dryland Stone Terraced System

A total of 123 medicinal plant species belonging to 51 families were reported to be used for treating human ailments in SDSTS (Table 1). Among them, 38 plant families were represented by one or two species while 13 families were represented by three species or more. Asteraceae was the most represented family with 16 species, followed by Fabaceae, Lamiaceae and Ranunculaceae with 8 species, respectively. Of the total, *Bupleurum chinense* DC., *Bupleurum scorzonerifolium* Willd., *Periploca sepium* Bunge, *Anemarrhena asphodeloides* Bunge, *Cirsium arvense* var. *integrifolium* C. Wimm. et Grabowski, *Taraxacum mongolicum* Hand. -Mazz., *Adenophora stricta* Miq., *Vigna radiata* (L.) Wilczek, *Scutellaria baicalensis* Georgi, *Forsythia suspensa* (Thunb.) Vahl, *Polygala tenuifolia* Willd., *Rumex crispus* L., *Agrimonia pilosa* Ledeb. and *Zanthoxylum bungeanum* Maxim. are the most frequently used species in the study area.

**Table 1:** Inventory of medicinal plants traditionally used by the local people in Wangjinzhuang Community

Family	Scientific name	Chinese name	Frequency	Habit	Habitat	Parts used	Preparations and uses
Adoxaceae	*Sambucus williamsii* Hance.	Jiegumu接骨木	*	Tree	Wild	Whole plant	Decoction; taken orally for hemostasis and promoting blood circulation, promoting reunion of bone and dispelling wind, draining dampness, treating for fracture, traumatic injury, rheumatic arthritis, gout, kashin-beck disease, chronic nephritis; Pounded fresh part applied on the affected area for traumatic bleeding
Amaranthaceae	*Achyranthes bidentata* Blume	Niuxi牛膝	*	Herb	Wild	Root	Decoction; taken orally for activating blood, promoting menstruation, liver and kidney, diuresis, treating for postpartum abdominal pain, irregular menstruation, hemorrhinia, kidney deficiency, amenorrhea
Amaranthaceae	*Bassia scoparia *(L.) A.J.Scott	Difu地肤	**	Herb	Wild	Whole plant	Decoction; taken orally for clearing heat and draining dampness, diuresis; treating for dysuria, gonorrhea, sexual impotence; Pounded fresh part applied on the affected area for bite, furuncle, scabies, rubella
Amaryllidaceae	*Allium macrostemon* Bunge	Xiebai薤白	**	Herb	Wild	Bulb	Decoction; taken orally for eliminating stagnation, analgesia effect, antibacterial effect, anti-inflammatory effect, antiviral effect, anti-tumor effect, reducing blood glucose and lipid, reducing cholesterol, tonifying Yang, anticoagulation and immune effect, preventing diabetes, preventing atherosclerosis and cerebral infarction, treating for asthma, stable angina, coronary heart disease, acute or chronic bronchitis, hyperlipoidemia, diarrhea, dysentery
Amaryllidaceae	*Allium ramosum* L.	Yejiu野韭	*	Herb	Wild	Seed	Decoction; taken orally for controlling nocturnal emission, promoting kidney and tonifying Yang, antibacterial effect, anti-inflammatory effect, antiviral effect, anti-tumor effect, analgesia and immune effect, antioxidation and antimutagenesis, treating for toothache, enuresis, hiccup, indigestion, gastric cavity
Anacardiaceae	*Pistacia chinensis* Bunge	Huanglianmu黄连木	*	Tree	Wild	Bark, leaf	Decoction; taken orally for clearing heat and detoxicating, draining dampness, moisturizing, anti-aging effect, treating for dysentery, psoriasis, hemorrhoids, stranguria, rheumatism sores, dermatitis rhus, pyogenic infections
Apiaceae	*Bupleurum chinense* DC.	Beichaihu北柴胡	***	Herb	Wild	Root	Decoction; taken orally for clearing heat, promoting blood circulation and regulating the flow of vital energy, promoting liver, strengthening spleen and kidney, relieving depression, tonifying Yang, treating for wind-heat cold and fever, liver qi stagnation, abnormal menstruation, rectocele, rheumatic arthritis, epigastric pain, liver depression and spleen deficiency, gastroenteritis, dysmenorrhea
Apiaceae	*Bupleurum scorzonerifolium* Willd.	Hongchaihu红柴胡	***	Herb	Wild	Root	Decoction; taken orally for clearing heat, promoting blood circulation and regulating the flow of vital energy, promoting liver, strengthening spleen and kidney, relieving depression, tonifying Yang, treating for wind-heat cold and fever, liver qi stagnation, abnormal menstruation, rectocele, rheumatic arthritis, epigastric pain, liver depression and spleen deficiency, gastroenteritis, dysmenorrhea
Apiaceae	*Foeniculum vulgare* Mill.	Huixiang茴香	*	Herb	Wild	Fruit	Decoction; taken orally for regulating the flow of vital energy, analgesia effect, antibacterial and anti-inflammatory effect, promoting stomach and spleen, promoting gastrointestinal motility, strengthening liver, treating for cold hernia and abdominal pain, dysmenorrhea, epigastric distension, sagging of one testicle, inappetence
Apocynaceae	*Vincetoxicum atratum *(Bunge) Morren et Decne.	Baiwei白薇	*	Herb	Wild	Root, stem	Decoction; taken orally for clearing heat and cooling blood, diuresis and detoxicating, anti-tumor and anti-inflammatory effect, treating for syncope, lymphangitis
Apocynaceae	*Cynanchum chinense* R. Br	Erongteng鹅绒藤	*	Liana	Wild	Whole plant	Decoction; taken orally for clearing heat and detoxicating, dispelling wind and analgesia effect, promoting stomach, detumescence, treating for retention of food, diarrhea due to damp-heat, verruca vulgaris
Apocynaceae	*Cynanchum rostellatum *(Turcz.) Liede & Khanum	Luomo萝藦	*	Liana	Wild	Whole plant	Decoction; taken orally for tonifying Yang, promoting kidney, regulating the flow of vital energy, hemostasis, detumescence and detoxification, analgesia and anti-tumor effect, moistening the lung and relieving asthma, treating for bronchitis and pneumonia, lactagogue method, leucorrhea, spermatorrhea, cough and asthma, impotence; Pounded fresh part applied on the affected area for pyogenic infections, bite, herpes zoster
Apocynaceae	*Periploca sepium* Bunge	Gangliu杠柳	***	Shrub	Wild	Bark	Decoction; taken orally for promoting muscles and bones, detumescence and draining dampness, analgesia and anti-tumor effect, dispelling wind, diuresis, treating edema of lower limbs, palpitation and short of breath, rheumatalgia, soreness and weakness of waist and knees, rheumatoid arthritis, dysuria
Araceae	*Pinellia ternata* (Thunb.) Breit.	Banxia半夏	**	Herb	Wild	Tuber	Decoction; taken orally for dissolving distension, arresting cough and eliminating phlegm, eliminating stagnation, antibacterial effect, anti-inflammatory effect, antiviral effect, anti-tumor effect, anticoagulation, preventing emesis, antioxidation, insecticidal effect, treating for cough due to excessive phlegm, epigastric distension and depression, headache dizziness, ulcer and pyogenic infections, nausea and vomiting; Pounded fresh part applied on the affected area for acute mastitis, suppurative otitis media
Aristolochiaceae	*Aristolochia debilis* Sieb. et Zucc.	Madouling马兜铃	*	Liana	Wild	Whole plant	Decoction; taken orally for promoting blood circulation and relieving pain, diuresis, treating for cough, chronic bronchitis, rheumatoid arthritis, hypertension, swelling and pain in throat, toothache
Aristolochiaceae	*Asarum heterotropoides* Fr. Schmidt	Xixin细辛	**	Herb	Wild	Whole plant	Decoction; taken orally for dispelling wind and cold, promoting blood circulation and detoxicating, analgesic effect and relieving asthma, treating for cough and asthma, wind-cold headache, hyperthermia and abdominal pain, traumatic injury, edema; Pounded fresh part applied on the affected area for snakebite
Asparagaceae	*Anemarrhena asphodeloides* Bunge	Zhimu知母	***	Herb	Wild	Rhizome	Decoction; taken orally for clearing heat, purging intense heat, quenching thirst and relieving restlessness, nourishing Yin and moistening dryness, anti-inflammatory effect, anti-tumor effect, reducing blood glucose and lipid, anti-thrombus, treating for Alzheimer's disease, fever with thirst, cough due to heat in lungs, deficiency of liver-yin and kidney-yin, intestinal dryness with constipation
Asparagaceae	*Asparagus cochinchinensis* (Lour.) Merr.	Tianmendong天门冬	*	Herb	Wild	Tuber	Decoction; taken orally for nourishing Yin and moistening dryness, moistening the lung and engendering liquid, anti-inflammatory effect, anti-tumor effect, reducing blood glucose and lipid, anti-thrombus, treating for dry throat and thirst, intestinal dryness with constipation, dry cough, phlegm, pulmonary tuberculosis, bronchitis, diphtheria, pertussis, diabetes, cardiovascular and cerebrovascular diseases; Pounded fresh part applied on the affected area for sore and ulcer, pyogenic infections, snakebite
Asparagaceae	*Polygonatum odoratum* (Mill.) Druce	Yuzhu玉竹	**	Herb	Wild	Rhizome	Decoction; taken orally for moistening the lung, nourishing Yin, hemostasis, promoting spleen and stomach, promoting kidney, protecting heart and bones, antibacterial effect, anti-tumor effect, analgesia and immune effect, anti-fatigue effect, anti-aging effect and antioxidation, reducing blood glucose and lipid, helping digestion, treating for cough without phlegm, dry throat and thirst, deficiency of Yin
Asparagaceae	*Polygonatum sibiricum* Delar. ex Redoute	Huangjing黄精	**	Herb	Wild	Rhizome	Decoction; taken orally for moistening the lung, nourishing Yin, hemostasis, promoting spleen and stomach, promoting kidney, anti-diabetic and anti-Alzheimer's disease effect, protecting heart and bones, antibacterial effect, anti-tumor effect, analgesia and immune effect, anti-fatigue effect, reducing blood glucose and lipid, treating for fracture, spleen and stomach qi deficiency, chronic hepatitis, inappetence, neurodermitis, acne vulgaris, chloasma, psoriasis, osteoporosis
Asparagaceae	*Ophiopogon japonicas* (L. f.) Ker-Gawl.	Maidong麦冬	*	Herb	Wild	Tuber	Decoction; taken orally for clearing heat and resolving phlegm, stopping emesis, nourishing Yin and engendering liquid, moistening the lung and calming the nerves, protecting cardiovascular system, anti-aging and anti-tumor effect, anti-inflammatory effect, treating for heart disease damaging liquid, thirsty and upset, insomnia with restlessness, intestinal dryness with constipation, dry cough, swelling and pain in throat, cough and asthma
Asphodelaceae	*Hemerocallis citrina* Baroni	Huanghuacai黄花菜	*	Herb	Wild	Whole plant	Decoction; taken orally for promoting spleen and stomach, promote lactation, replenishing blood, calming the nerves, diuresis and detumescence, preventing gastrointestinal cancer, treating for hypogalactia, edema and dysuria, neurasthenia, insomnia with restlessness, out of lunch, forgetfulness, hypertension
Asphodelaceae	*Hemerocallis fulva* (L.) L.	Xuancao萱草	**	Herb	Wild & cultivated	Whole plant	Decoction; taken orally for clearing heat and draining dampness, diuresis and cooling blood, hemostasis, antibacterial effect, anti-inflammatory effect, insecticidal effect, anti-tumor effect, antioxidation, treating for edema, dysuria, stranguria, morbid leukorrhea, jaundice, hematochezia and metrorrhagia and metrostaxis, acute mastitis, breast milk stoppage, scrofula
Asteraceae	*Arctium lappa* L.	niubang牛蒡	**	Herb	Wild	Fruit, root, leaf, flower	Decoction; taken orally for clearing heat and detoxicating, detumescence and removing phlegm, analgesia effect, promoting eruption and relieving sore throat, treating for diabetes, hyperlipidemia, hypertension, wind-heat common cold, swelling and pain in throat, cough, atherosclerosis; Pounded fresh part applied on the affected area for mammary pain and pruritus, headache
Asteraceae	*Artemisia annua* L.	Huanghuahao黄花蒿	*	Herb	Wild	Whole plant	Decoction; taken orally for clearing heat and detoxicating, diuresis and cooling blood, treating for night sweat, malaria, heatstroke, cold; Pounded fresh part applied on the affected area for malignant sore, scabies
Asteraceae	*Artemisia argyi* Levl. et Van	Aihao艾蒿	*	Herb	Wild	Whole plant	Decoction; taken orally for clearing heat and detoxicating, hemostasis, dispelling cold and removing dampness, relieving asthma and cough, miscarriage prevention, anti-inflammatory and antiallergic effect, treating for dysmenorrhea, chronic bronchitis and asthma; Washing in soup for infection of maternal and infant diseases in delivery period; Hanging on doors as antibiosis and repellents
Asteraceae	*Artemisia capillaries* Thunb.	Yinchenhao茵陈蒿	*	Herb	Wild	Whole plant	Decoction; taken orally for clearing heat and detoxicating, treating for jaundice
Asteraceae	*Artemisia caruifolia *Buch. -Ham. ex Roxb.	Qinghao青蒿	*	Herb	Wild	Whole plant	Decoction; taken orally for clearing heat and clearing summer heat, diuresis and miscarriage prevention, hemostasis and cooling blood, treating for cough due to heat in lungs, swelling and pain in throat, jaundice, malaria, gonorrhea, hematemesis, rheumatalgia, hemoptysis, bleeding due to external injury, malignant sore
Asteraceae	*Atractylodes lancea* (Thunb.) DC.	Cangzhu苍术	**	Herb	Wild	Rhizome	Decoction; taken orally for dispelling cold and wind, removing dampness, promoting eyesight and spleen, analgesia effect, immunomodulatory effect, anti-inflammatory effect, anti-tumor effect, promoting liver, treating for gastric ulcer, epigastric distension and depression, center burner damp obstruction, nyctalopia
Asteraceae	*Bidens parvifiora* Willd.	Guizhencao小花鬼针草	*	Herb	Wild	Whole plant	Decoction; taken orally for clearing heat and detoxicating, promoting blood circulation and dissipating blood stasis, analgesia effect, antibacterial and anti-inflammatory effect, anti-tumor effect, promoting liver, reducing blood sugar and blood lipids, treating for infection of the upper respiratory tract, swelling and pain in throat, hypertension, acute appendicitis, acute icteric hepatitis, gastroenteritis, rheumatalgia, arthralgia and malaria; Pounded fresh part applied on the affected area for snakebite, furuncle, traumatic injury
Asteraceae	*Bidens pilosa* L.	Guizhencao鬼针草	**	Herb	Wild	Whole plant	Decoction; taken orally for clearing heat and detoxicating, detumescence, dispersing blood stasis, analgesia effect, antibacterial and anti-inflammatory effect, anti-tumor effect, promoting liver, reducing blood sugar and blood lipids, treating for malaria, diarrhea, hepatitis, dysentery, acute nephritis, stomachache, dysphagia, intestinal carbuncle, swelling and pain in throat, traumatic injury, hypertension, diabetes, coronary heart disease, tracheophyma, xerophthalmia, chronic bronchitis, emphysema and neurasthenia; Pounded fresh part applied on the affected area for snakebite, furuncle
Asteraceae	*Carpesium cernuum* L.	Yanguantoucao烟管头草	*	Herb	Wild	Whole plant	Decoction; taken orally for clearing heat and detoxicating, removing phlegm, preventing malaria, antibacterial and anti-inflammatory effect, anti-tumor effect, promoting liver, treating for toothache, malaria, laryngalgia; fumigating and washing in soup for scabies, impetigo, hemorrhoids
Asteraceae	*Cirsium japonicum* Fisch. ex DC.	Daji大蓟	**	Herb	Wild	Leaf, root	Decoction; taken orally for hemostasis and cooling blood, eliminating blood stasis and carbuncles, detoxicating, reducing blood sugar, anti-tumor effect, protecting liver and antioxidation, treating for acute tonsillitis, pulmonary tuberculosis, stenocardia, hypertension, myocardial infarction, high-cholesterol, traumatic injury, diabetes, abscess and sore toxin, intestinal carbuncle, traumatic hemorrhage, hemoptysis, hematuria, metrorrhagia and metrostaxis
Asteraceae	*Cirsium arvense* var.* integrifolium *C. Wimm. et Grabowski	Ciercai刺儿菜	***	Herb	Wild	Leaf, root	Decoction; taken orally for hemostasis and cooling blood, dissipating blood stasis, detumescence and detoxicating, antibacterial effect, anti-inflammatory effect, preventing gastric mucosal lesion, reducing blood sugar, anti-tumor effect, protecting liver and antioxidation, treating for traumatic hemorrhage, acute tonsillitis, pulmonary tuberculosis, hemoptysis, hematuria, metrorrhagia and metrostaxis
Asteraceae	*Erigeron canadensis* L.	Xiaofeipeng小飞蓬	*	Herb	Wild	Whole plant	Decoction; taken orally for clearing heat and detoxicating, diuresis, antibacterial and anti-inflammatory effect, hemostasis, treating for enteritis, dysentery, infectious hepatitis and cholecystitis, rheumatalgia
Asteraceae	*Takhtajaniantha austriaca *(Willd.) Zaika, Sukhor. & N. Kilian	Yacong鸦葱	*	Herb	Wild	Whole plant	Decoction; taken orally for clearing heat and detoxicating, lactagogue method, dispelling wind and removing dampness, regulating the flow of vital energy, promoting blood circulation, diuresis, antibacterial and analgesia effect, anti-inflammatory effect, antiviral and anti-stress effect, anti-tumor effect, antioxidation, promoting liver, anti-depression effect, reducing blood lipids, treating for pernicious vomiting
Asteraceae	*Senecio scandens* Buch. -Ham.	Qianliguang千里光	*	Herb	Wild	Whole plant	Decoction; taken orally for clearing heat and detoxicating, detumescence, removing nebula for improving eyesight, dissipating blood stasis, relieving itching, insecticidal effect, antibacterial and analgesia effect, anti-inflammatory effect, antiviral and anti-trichomonal effect, anti-tumor effect, antioxidation, promoting liver, treating for trichomonas vaginalis, wind-heat common cold, swelling and pain of eye, diarrhea and dysentery, eczema, infection of the upper respiratory tract, acute tonsillitis, swelling and pain in throat, pneumonia
Asteraceae	*Taraxacum mongolicum* Hand. -Mazz.	Pugongying蒲公英	***	Herb	Wild	Whole plant	Decoction; taken orally for clearing heat and detoxicating, detumescence, removing stasis, diuresis and treating stranguria, cholagogue, anti-radiation, anti-fatigue effect, antibacterial effect, anti-inflammatory effect, anti-tumor effect, promoting liver, promoting the resumption of gastrointestinal function, treating for acute upper respiratory tract infection, diabetes, ulcer, subacute eczema, demodicidosis, gastric cancer, climacteric syndrome, abdominal pain, milk withdrawal, infertility, external hemorrhoid, suppurative otitis media, erysipelas, leucorrhea; Pounded fresh part applied on the affected area for acute mastitis
Asteraceae	*Xanthium sibiricum* Patrin. ex Widder.	Canger苍耳	*	Herb	Wild	Whole plant, fruit	Whole plant: Decoction; taken orally for clearing heat and detoxicating, removing wind dampness, dituesis, antiallergic effect, treating for chronic rhinitis, acute parasinusitis, leprosy, ileotyphus, skin cancer, functional uterine bleeding, rheumatoid arthritis, rheumatic heart disease, urticaria, allergic asthma; Fruit: treating for wind-cold headache, epistaxis, rubella and pruritus
Bignoniaceae	*Incarvillea sinensis* Lam.	Jiaohao角蒿	*	Herb	Wild	Whole plant	Decoction; taken as a water bath or pounded fresh part applied on the affected area for promoting blood circulation, regulating menstruation, anti-inflammatory and analgesia effect, treating for hepatitis, stomachache, abnormal menstruation, hypertension, gall of fracture, dizziness, anemia, calculus
Brassicaceae	*Raphanus sativus* L.	Luobo萝卜	**	Herb	Cultivated	Seed	Decoction; taken orally for promoting digestion and eliminating flatulence, resolving phlegm, treating for constipation, chronic bronchitis, chronic lung disease, halitosis and traumatic injury
Campanulaceae	*Adenophora stricta* Miq.	Shashen沙参	***	Herb	Wild	Root	Decoction; taken orally for nourishing Yin, moistening lung, clearing heat and resolving phlegm, cooling blood, regulating the flow of vital energy, anti-ulcer and anti-tumor effect, immunoregulatory function and antioxidation, anti-radiation effect, improving learning and memory, antifungal effect, treating for cough due to heat in lungs, tussiculation due to phlegm
Campanulaceae	*Codonopsis pilosula* (Franch.) Nannf.	Dangshen党参	*	Herb	Wild	Root	Decoction; taken orally for engendering liquid and nourishing blood, anti-inflammatory and anti-tumor effect, antioxidation and promoting liver, benefiting for spleen and lung, decreasing blood lipids, treating for heart failure, palpitations, shortness of breath, panasthenia, asthenia of qi and blood, anemia
Campanulaceae	*Platycodon grandiflorus* (Jacq.) A. DC.	Jiegeng桔梗	**	Herb	Wild	Root	Decoction; taken orally for moistening lung and engendering liquid, relieving asthma and relieving a cough, promoting spleen and lung, anti-inflammatory and anti-tumor effect, treating for cough due to excessive phlegm, swelling and pain in throat, thoracic fullness and rib-side pain, pulmonary abscess, dysentery and abdominal pain, dysuria
Cannabaceae	*Humulus scandens* (Lour.) Merr.	Lücao葎草	*	Herb	Wild	Whole plant	Decoction; taken orally for clearing heat and detoxicating, diuresis, treating heat stranguria, treating for cough due to heat in lungs, dysuria; Pounded fresh part applied on the affected area for edema, pruritus, carbuncle
Caprifoliaceae	*Lonicera japonica* Thunb.	Rendong忍冬	*	Liana	Wild	Flower, stem, fruit	Decoction; taken orally for clearing heat and detoxicating, detumescence, antibacterial and anti-inflammatory effect, anti-tumor effect, analgesic effect and antioxidation, antiviral and antiallergy effect, benefiting for early pregnancy, reducing blood sugar and blood lipid, treating for dysentery with bloody stool, sore and furuncle
Celastraceae	*Celastrus orbiculatus* Thunb	Nansheteng南蛇藤	*	Shrub	Wild	Stem, leaf, root, seed, fruit	Decoction; taken orally for clearing heat and detoxicating, calming the nerves, promoting blood circulation, dispelling wing and removing dampness, detumescence, anti-inflammatory and anti-tumor effect, antioxidation, treating for arthralgia and myalgia, lumbago, rheumatic arthritis, toothache, amenorrhea, dysentery; Pounded fresh part applied on the affected area for traumatic injury, abscess, furuncle and carbuncle, snakebite, eczema
Celastraceae	*Euonymus alatus* (Thunb.) Sieb.	Weimao卫矛	*	Shrub	Wild	Stem, leaf, root, fruit	Decoction; taken orally for clearing heat and detoxicating, anti-inflammatory and antibacterial effect, dispelling wind and relieving pain, dissipating blood stasis, regulating menstruation, hemostasis, promoting blood circulation, treating for hypertension and hyperlipidemia, metrorrhagia and metrostaxis, abdominal distension and pain, diabetes, coronary heart disease, nephrosis, hernia; Pounded fresh part applied on the affected area for traumatic injury, snakebite, rheumatalgia, dermatitis
Convolvulaceae	*Cuscuta chinensis* Lam.	Tusizi菟丝子	*	Herb	Wild	Seed	Decoction; taken orally for tonifying Yang, miscarriage prevention, promoting liver and kidney, improving eyesight, anti-aging and immunomodulatory effect, treating for lactagogue method, impotence, diabetes, spermatorrhea, kidney deficiency, threatened abortion; Pounded fresh part applied on the affected area for leucoderma
Crassulaceae	*Phedimus aizoon *(L.)’t Hart	Feicai费菜	**	Herb	Wild	Whole plant	Decoction; taken orally for clearing heat and detoxicating, dissipating blood stasis, hemostasis, tranquilization, treating for hemoptysis, hematemesis, hematochezia, hematuria, palpitation and insomnia; Pounded fresh part applied on the affected area for traumatic injury, sore and furuncle, carbuncle and burn
Crassulaceae	*Sedum sarmentosum* Bunge	Chuipencao垂盆草	*	Herb	Wild	Whole plant	Decoction; taken orally for clearing heat and detoxicating, diuresis and detumescence, apocenosis and promoting tissue regeneration, anti-inflammatory effect, treating for jaundice, dysuria, hepatitis, pancreatitis and swelling and pain in throat; Pounded fresh part applied on the affected area for sore and furuncle, carbuncle
Dioscoreaceae	*Discorea nipponica* Makino	Chuanlongshuyu穿龙薯蓣	*	Liana	Wild	Rhizome	Decoction; taken orally for resolving phlegm, antibacterial effect, anti-inflammatory effect, antiviral effect, anti-tumor effect, immune effect, antioxidation, reducing blood glucose, promoting liver and kidney, treating for coronary heart disease, stable angina, rheumatic arthritis, chronic bronchitis, bronchitic asthma, diabetes, thyroid adenoma, acute cerebral infarction
Dioscoreaceae	*Dioscorea polystachya *Turczaninow	Shuyu薯蓣	*	Liana	Wild	Rhizome	Decoction; taken orally for antibacterial effect, anti-inflammatory effect, antiviral effect, anti-tumor effect, immune effect, antioxidation, reducing blood glucose, promoting liver and kidney, reducing phlegm, treating for diarrhea, dysentery, seminal discharge and amnesia
Ebenaceae	*Diospyros kaki *Thunb.	Shishu柿树	*	Tree	Cultivated	Root, fruit, leaf	Decoction; taken orally for clearing heat and cooling blood, moistening lung and engendering liquid, hemostasis and reducing blood pressure, treating for cough due to lung heat, swelling and pain in throat, gastrointestinal hemorrhage, hypertension, bleeding from hemorrhoids, dysentery with bloody stool
Ebenaceae	*Diospyros lotus* L.	Junqianzi君迁子	*	Tree	Wild	Leaf, fruit, seed	Decoction; taken orally for promoting spleen and stomach, nourishing the blood and calming the nerves, antibacterial and anti-AIDS effect, increasing immunity, nourishing yin, improving digestion, preventing osteoporosis and postpartum anemia, treating for hypertension, senile cataract, premature graying, cough
Fabaceae	*Albizia julibrissin* Durazz.	Hehuan合欢	*	Tree	Wild	Flower, bark	Decoction; taken orally for resolving depression, calming the nerves and promoting appetite, antibacterial effect, treating for insomnia, depression, obesity, pulmonary abscess; Pounded fresh part applied on the affected area for traumatic injury
Fabaceae	*Caragana sinica* (Buc'hoz) Rehd.	Jinji’er锦鸡儿	*	Shrub	Wild	Flower	Decoction; taken orally for dispelling wind and relieving pain, expelling phlegm and relieving a cough, promoting spleen and stomach, clearing heat and detumescence, anti-inflammatory and antibacterial effect, anti-tumor effect and antioxidation, analgesic effect, treating for rheumatalgia, arthralgia, edema due to spleen deficiency, infantile malnutrition
Fabaceae	*Gleditsia japonica* Miq.	Shanzaojia山皂荚	**	Tree	Wild	Seed, fruit, bark, thorn	Decoction; taken orally for relaxing bowels, dispelling wind and detumescence, eliminating phlegm, treating for constipation, hernia, cough; Pounded fresh part applied on the affected area for scrofula, sore and tinea, abscess
Fabaceae	*Gleditsia sinensis* Lam.	Zaojia皂荚	*	Tree	Wild	Thorn	Decoction; taken orally for expelling toxins and detumescence, apocenosis, anti-tumor effect, treating for cancer
Fabaceae	*Vigna radiata *(L.) Wilczek	Lüdou绿豆	***	Herb	Wild	Seed	Decoction; taken orally for detoxicating, promoting liver, antibacterial and anti-tumor effect, nourishing skin, treating for indigestion, hypertension, diabetes, nephritis, heatstroke, urinary tract infection, chronic prostatitis, cephalalgia, eczema, parotitis; Pounded fresh part applied on the affected area for traumatic injury, dysentery, swelling
Fabaceae	*Sophora flavescens* Alt.	Kushen苦参	**	Herb	Wild	Root	Decoction; taken orally for clearing heat and detoxicating, anti-inflammatory and analgesic effect, anti-tumor effect, bacteriostasis and diuresis, anti-hepatic fibrosis, treating for heat dysentery, hematochezia, chronic hepatitis B, chronic hepatitis C, eczema and arrhythmia, abnormal leukorrhea, pruritus of vagina; Pounded fresh part applied on the affected area for vaginosis
Fabaceae	*Vicia amoena* Fisch.	Shanyewandou山野豌豆	**	Herb	Wild	Whole plant	Decoction; taken orally for dispelling wind and removing dampness, promoting blood circulation and analgesia, treating for functional uterine hemorrhage, epistaxis; Fumigating and washing the affected area for rheumatalgia, eczema; Pounded fresh part applied on the affected area for fall damage, unknown pyogenic infections
Fabaceae	*Vigna umbellata* (Thunb.) Ohwi et Ohashi	Chixiaodou赤小豆	*	Herb	Cultivated	Seed	Decoction; taken orally for diuresis, detumescence, treating stranguria, promoting digestion and spleen, replenishing blood and removing dampness, treating for acute nephritis, cirrhotic ascites, jaundice and crystalli, mumps
Gentianaceae	*Gentiana squarrosa* Ledeb.	Linyelongdan鳞叶龙胆	*	Herb	Wild	Root, rhizome	Decoction; taken orally for clearing heat and draining dampness, promoting digestion, reducing blood pressure, clearing liver fire, anti-inflammatory and antiallergic effect, anticonvulsant effect, treating for acute icteric infectious hepatitis, damp-heat jaundice, leucorrhea, epilepsy, pruritus of private parts, infantile malnutrition, swelling and pain in throat and eczema
Geraniaceae	*Erodium stephanianum* Willd.	Mangniuermiao牻牛儿苗	*	Herb	Wild	Whole plant	Decoction; taken orally for clearing heat and detoxicating, dredging collaterals, promoting blood circulation, treating for arthralgia and myalgia, numb hands and feet, dysuria, hemiplegia, diarrhea
Iridaceae	*Belamcanda chinensis* (L.) DC	Shegan射干	**	Herb	Wild	Rhizome	Decoction; taken orally for clearing heat and detoxicating, dissipating blood stasis and detumescence, dissolving phlegm, benefiting throat, antibacterial effect, anti-inflammatory effect, eliminating stagnation, treating for influenza, infection of the upper respiratory tract, swelling and pain in throat and cough
Iridaceae	*Iris dichotoma* pall.	Yeyuanwei野鸢尾	*	Herb	Wild	Rhizome	Decoction; taken orally for removing food retention, dissipating blood stasis and detumescence, detoxicating, antibacterial effect, anti-inflammatory effect, antiviral effect, anti-tumor effect, treating for arthritis, swelling and pain in throat, hepatitis, dyspepsia, bronchitis and traumatic injury; Pounded fresh part applied on the affected area for dermatitis
Juglandaceae	*Juglans regia *L.	Hutao胡桃	*	Tree	Wild	Kernel	Taken as nut or cooked with meat for benefiting qi and nourishing blood, calming nerves, relaxing the bowels, promoting kidney and brain, treating for neurasthenia, emission due to the kidney deficiency, frequent micturition and amnesia
Lamiaceae	*Ajuga ciliata* Bunge	Jingucao筋骨草	**	Herb	Wild	Whole plant	Decoction; taken orally for clearing heat and detoxicating, cooling blood and detumescence, analgesia and anti-inflammatory effect, antibacterial and anti-tumor effect, treating for swelling and pain in throat, hemoptysis due to lung heat, chronic bronchitis, chronic glomerulonephritis, hepatitis, acute pneumonia; Pounded fresh part applied on the affected area for traumatic injure, traumatic bleeding
Lamiaceae	*Elsholtzia ciliata* (Thunb.) Hyland.	Xiangru香薷	*	Herb	Wild	Whole plant	Decoction; taken orally for clearing heat and detoxicating, relieving exterior syndrome by diaphoresis, analgesia and anti-inflammatory effect, diuresis and detumescence, treating for acute gastroenteritis, vomiting and diarrhea, abdominal pain, cholera, hyperthermia, epistaxis, edema, barbiers and halitosis
Lamiaceae	*Lagopsis supina* (Steph. ex Willd.) Ik. -Gal. ex Knorr.	Xiazhicao夏至草	*	Herb	Wild	Whole plant	Decoction; taken orally for promoting blood circulation, removing blood stasis, regulating menstruation, treating for abnormal menstruation, hemiplegia, amenorrhea, anemia
Lamiaceae	*Leonurus japonicus* Houttuyn	Yimucao益母草	**	Herb	Wild	Whole plant	Decoction; taken orally for clearing heat and detoxicating, diuresis and improving eyesight, promoting blood circulation, removing blood stasis, regulating menstruation, detumescence, treating for leukorrhea, prolapse of uterus, metrorrhagia, dysmenorrhea, dysuria, edema, hypertension, abnormal menstruation, hemiplegia, amenorrhea, anemia
Lamiaceae	*Salvia miltiorrhiza* Bunge	Danshen丹参	**	Herb	Wild	Root	Decoction; taken orally for promoting blood circulation, removing blood stasis, regulating menstruation, nourishing blood for tranquillization, analgesic effect, cooling blood, treating for ulcer and carbuncle, angina pectoris, irregular menstruation, amenorrhea, postpartum blood stasis and abdominal pain, insomnia, emesis, cough due to heat in lungs, kidney deficiency and mild lumbago, traumatic injury, dysmenorrhea
Lamiaceae	*Scutellaria baicalensis* Georgi	Huangqin黄芩	***	Herb	Wild	Root	Decoction; taken orally for clearing heat and purging fire, hemostasis and anti-inflammatory effect, antibacterial and anti-tumor effect, antioxidation, preventing cardiovascular and cerebrovascular disease, diuresis, promoting kidney, treating for hemoptysis, enteritis, dysentery, jaundice, hypertension, cold, headache due to wind-heat, oppression in chest, lung heat, pneumonia, threatened abortion
Lamiaceae	*Vitex negundo* L. var. *heterophylla* (Franch.) Rehd.	Jingtiao荆条	*	Shrub	Wild	Whole plant	Decoction; taken orally for relieving exterior syndrome, resolving dampness, analgesia and insecticidal effect, clearing heat and detoxicating, relieving a cough and asthma, immunomodulatory and anti-early pregnancy effect, treating for common cold due to wind-cold, acute gastroenteritis, dysentery chronic bronchitis, malaria, gonorrhea, enterobiasis, stomachache, toothache; washing in soup for dermatitis, eczema, porrigo
Lamiaceae	*Schnabelia terniflora* (Maxim.) P. D. Cantino	Sanhuayou三花莸	*	Shrub	Wild	Whole plant	Decoction; taken orally for relieving exterior syndrome and dispelling cold, facilitating lung, treating for headache, cough, nebula and burn
Liliaceae	*Lilum browaii* var.* viridulum* Baker	Baihe百合	*	Herb	Wild	Bulb	Decoction; taken orally for moistening the lung, nourishing Yin, hemostasis, relieving cough and asthma, removing phlegm, calming the nerves, anti-depression, anti-tumor effect, analgesia and immune effect, anti-fatigue and antioxidation, reducing blood glucose, treating for cough and asthma due to heat in lungs, cough with blood-flecked phlegm, pulmonary abscess, senile chronic bronchitis, neurasthenia, palpitations, sleeplessness
Malvaceae	*Abutilon theophrasti* Medicus	Qingma苘麻	*	Herb	Wild	Seed	Decoction; taken orally for clearing heat and detoxicating, draining dampness, removing nebula, treating for dysentery, stranguria, pyogenic infections
Menispermaceae	*Menispermum dauricum* DC.	Bianfuge蝙蝠葛	*	Liana	Wild	Root, stem	Decoction; taken orally for clearing heat and detoxification, anti-inflammatory and analgesic effects, dispelling wind, relieving pain, treating for enteritis, dysentery, rheumatic arthralgia, amygdalitis, swelling and pain in throat, cerebrovascular diseases
Moraceae	*Broussonetia papyrifera* (L.) L'Heritier ex Ventenat	Gou构	*	Tree	Wild	Bark, root, fruit	Decoction; taken orally for clearing heat and removing dampness, cooling blood and insecticidal action, promoting eyesight, reinforcing kidney, treating for sexual impotence, dysentery, enteritis, soreness and weakness of waist and knees; Pounded fresh part applied on the affected area for neurodermatitis, tinea
Moraceae	*Morus alba* L.	Sang桑	*	Tree	Wild	Leaf, root, stem, fruit, bark	Decoction; taken orally for clearing heat and dispelling the wind, promoting eyesight and clearing away the lung heat, treating for cough due to heat in lungs, trachitis, rheumatoid arthritis, diarrhea; Pounded fresh part applied on the affected area for traumatic injury, acariasis
Oleaceae	*Forsythia suspensa* (Thunb.) Vahl	Lianqiao连翘	***	Shrub	Wild	Fruit	Decoction; taken orally for clearing heat and detoxicating, strengthening heart, antibacterial and analgesia effect, anti-inflammatory and antiviral effect, promoting liver, treating for urinary obstruction, erythrogenic toxin, scrofula, acute mastitis
Oleaceae	*Fraxinus bungeana* DC.	Xiaoyecen小叶梣	*	Tree	Wild	Bark	Decoction; taken orally for clearing heat and draining dampness, diuresis, promoting liver and eyesight, antibacterial and analgesia effect, anti-inflammatory and antiviral effect, antiallergic and anti-tumor effect, antioxidation, calming the nerves, treating for enteritis, chronic bronchitis, dysentery, nebula, hot eyes
Oleaceae	*Syringa oblata* Lindl.	Zidingxiang紫丁香	*	Shrub	Wild	Leaf	Decoction; taken orally for clearing heat and quenching one's thirst, antibacterial and anti-inflammatory effect, promoting eyesight, treating for emesis, diarrhea, rheumatalgia, hernia, keratitis and conjunctivitis
Orobanchaceae	*Siphonostegia chinensis* Benth.	Yinxingcao阴行草(北刘寄奴)	**	Herb	Wild	Whole plant	Decoction; taken orally for clearing heat and draining dampness, promoting blood circulation, dissipating blood stasis, diuresis, antibacterial effect, anti-inflammatory effect, removing phlegm, relieving asthma and cough, reducing serum cholesterol and blood lipid, cholagogue and promoting liver, treating for acute and chronic icteric hepatitis, chronic bronchitis, cervical cancer, skin cancer, cholecystitis, dysuria, abdominal distension, postpartum abdominal pain and dysentery with bloody stool
Orobanchaceae	*Rehmannia glutinosa* (Gaert.) Libosch. ex Fisch. et Mey.	Dihuang地黄	**	Herb	Wild	Root, stem	Decoction; taken orally for clearing heat and cooling blood, promoting liver and heart, nourishing yin, promoting kidney, immunomodulatory effect, anti-tumor and anti-inflammatory effect, antibacterial effect, preventing osteoporosis, hemostasis, treating for spontaneous external bleeding, hemafecia, hematuria, hematemesis, hemoptysis, metrorrhagia and metrostaxis, retinal hemorrhage, depression, abnormal menstruation, keratomycosis, diabetes
Papaveraceae	*Chelidonium majus* L.	Baiqucai白屈菜	*	Herb	Wild	Whole plant	Decoction; taken orally for relieving a cough and pain, detoxification, bactericidal effect, diuresis, treating for fever, liver cirrhosis, beriberi, duodenal ulcer, gastritis, gastric ulcer; Pounded fresh part applied on the affected area for scabies, detumescence
Papaveraceae	*Corydalis racemosa* (Thunb.) Pers.	Xiaohuahuangjin小花黄堇	*	Herb	Wild	Whole plant	Decoction; taken orally for clearing heat and detoxicating, moistening lung, relieving a cough and itching, astringe effect; Pounded fresh part applied on the affected area for scabies, carbuncle, sore and furuncle, snakebite, stubborn dermatitis
Papaveraceae	*Dicranostigma leptopodum* (Maxim.) Fedde	Tuchuanghua秃疮花	*	Herb	Wild	Whole plant	Decoction; taken orally for clearing heat and detoxicating, anti-inflammatory effect, detumescence, relieving pain, insecticidal effect, treating for toothache; Washed with water for tinea, itch
Phrymaceae	*Phrym leptostachya* L. var.* asiatica* Hara	Tougucao透骨草	**	Herb	Wild	Whole plant	Decoction; taken orally for spawning induction, insecticidal effect, treating for dystocia; Pounded fresh part applied on the affected area for furuncle and carbuncle, swelling
Phyllanthaceae	*Leptopus chinensis* (Bunge) Pojark.	Queershetou雀儿舌头	*	Shrub	Wild	Root	Decoction; taken orally for regulating the flow of vital energy and relieving pain, promoting spleen and stomach, treating for stomachache, diarrhea, edema, jaundice, abdominal distension and pain, inappetence
Plantagianceae	*Plantago asiatica* L.	Cheqian车前	*	Herb	Wild	Whole plant	Decoction; taken orally for clearing heat and detoxicating, diuresis, promoting liver and eyesight, antibacterial and anti-inflammatory effect, reducing blood pressure, relieving a cough and phlegm, treating for asthma, cough due to phlegm, diarrhea, chronic active hepatitis, latent glomerulonephritis, pannus, dysentery and hematuria; Pounded fresh part applied on the affected area for decubitus, postpartum urinary retention, acute mastitis; Washing in soup for elytritis
Poaceae	*Setaria italica *var.* germanica *(Mill.) Schred.	Qinggu青谷	*	Herb	Cultivated	Fruit	Decoction; taken orally for promoting digestion, promoting spleen and stomach, diuresis, treating for retention of food, abdominal distension, halitosis, spleen-stomach deficiency, inappetence, constipation
Polygalaceae	*Polygala sibirica* L.	XiboliyaYuanzhi西伯利亚远志	*	Herb	Wild	Root	Decoction; taken orally for calming the nerves and dispelling depression, diuresis, promoting liver and heart, anti-senile dementia and anti-tumor effect, eliminating phlegm and detumescence, anti-inflammatory effect, improving memory, regulating blood sugar, treating for hypertension and neurasthenia, acute mastitis, insomnia and dreamful sleep, forgetfulness and pavor, dizziness, breast pain, sore and pyogenic infections
Polygalaceae	*Polygala tenuifolia* Willd.	Yuanzhi远志	***	Herb	Wild	Root	Decoction; taken orally for treating similar ailments as *Polygala sibirica*
Polygonaceae	*Rumex crispus* L.	Zhouyesuanmo皱叶酸模	***	Herb	Wild	Root	Decoction; taken orally for strengthening the kidney, diuresis, promoting circulation and hemostasis, diuresis, treating for pulmonary abscess, vomiting blood, constipation, indigestion; Pounded fresh part applied on the affected area for dermatitis, furuncle, scabies, eczema, burn
Pteridaceae	*Aleuritopteris argentea*（Gmel.）F´ee	Yinfenbeijue银粉背蕨	*	Herb	Wild	Whole plant	Decoction; taken orally for clearing heat and detoxicating, treating for hemostasis
Ranunculaceae	*Aconitum kusnezoffii* Rchb.	Beiwutou北乌头	*	Herb	Wild	Root	Decoction; taken orally for dispelling wind, relieving pain, removing dampness, treating for arthritis, neuralgia, toothache, stroke
Ranunculaceae	*Aconitum sinomontanum* Nakai	Gaowutou高乌头	*	Herb	Wild	Root	Decoction; taken orally for analgesia effect, removing phlegm, treating for palpitation, aphasia from apoplexy, wind-cold-dampness, arthralgia; Pounded fresh part applied on the affected area for traumatic injury
Ranunculaceae	*Anemone tomentosa* (Maxim.) Pei	Dahuocao大火草	*	Herb	Wild	Stem	Decoction; taken orally for reducing phlegm, dissipating blood stasis, insecticidal effect, clearing heat and detoxicating, treating for malaria, infantile malnutrition, cough asthma, dysentery; Pounded fresh part applied on the affected area for sore, furuncle, carbuncle, traumatic injury
Ranunculaceae	*Aquilegia viridiflora* Pall.	Loudoucai耧斗菜	**	Herb	Wild	Whole plant	Decoction; taken orally for clearing heat and detoxicating, promoting blood circulation and hemostasis, treating for abnormal menstruation, metrorrhagia and metrostaxis, dysmenorrhea and dysentery
Ranunculaceae	*Clematis heracleifolia* DC.	Dayetiexianlian大叶铁线莲	*	Herb	Wild	Whole plant	Decoction; taken orally for dispelling wind and eliminating dampness, clearing heat and detumescence, treating for rheumatic joint pains, tuberculous ulcer, fistula
Ranunculaceae	*Clematis kirilowii *Maxim.	Taihangtiexianlian太行铁线莲	*	Liana	Wild	Whole plant	Decoction; taken orally for detoxification, dredging channels and collaterals, diuresis, treating for digestion, urinary tract infection, constipation, breast milk stoppage, rheumatic arthritis, amenorrhea; Pounded fresh part applied on the affected area for bite, toothache, web-eye
Ranunculaceae	*Clematis intricata* Bunge	Huanghuatiexianlian黄花铁线莲	*	Liana	Wild	Whole plant	Pounded fresh part applied on the affected area for expelling wind-damp, treating for chronic rheumatoid arthritis, rash
Ranunculaceae	*Pulsatilla chinensis *(Bunge) Regel	Baitouweng白头翁	*	Herb	Wild	Stem	Decoction; taken orally for clearing heat and detoxicating, cooling blood and checking dysentery, draining dampness, insecticidal effect, treating for bloody flux, warm malaria, epistaxis, bleeding from hemorrhoids
Rhamnaceae	*Ziziphus jujuba* var. *spinosa* (Bunge) Hu ex H.F. Chow	Suanzao酸枣	**	Shrub	Wild	Whole plant	Decoction; taken orally for calming the nerves, promoting heart and liver, detoxicating, reducing blood pressure, softening blood vessel, treating for scurvy, insomnia, coronary heart disease, hypertension
Rosaceae	*Agrimonia pilosa* Ledeb.	Longyacao龙芽草	***	Herb	Wild	Whole plant	Decoction; taken orally for cooling blood and hemostasis, strengthening heart and promoting stomach, treating for hemoptysis, dysentery, hematuria, hemorrhage of internal lesion caused by overexertion
Rosaceae	*Crataegus cuneata* Sied. et. Zucc.	Yeshanzha野山楂	*	Shrub	Wild	Fruit	Decoction; taken orally for helping digestion and promoting stomach, promoting heart, dissipating blood stasis, softening blood vessel, decreasing hemorrheologic, treating for constipation, diarrhea, hypertension
Rosaceae	*Potentilla chinensis* Ser.	Weilingcai委陵菜	*	Herb	Wild	Whole plant	Taken orally for clearing heat and detoxicating, dispelling wind, treating for rheumatic pain, dysentery, paralysis, epilepsy
Rosaceae	*Potentilla discolor* Bge.	Fanbaicao翻白草	*	Herb	Wild	Whole plant	Taken orally for clearing heat and detoxicating, hemostasis and detumescence, treating for dysentery, cough, pulmonary abscess, hemoptysis, metrorrhagia and metrostaxis, abscess
Rosaceae	*Pyrus betulifolia* Bunge	Duli杜梨	**	Tree	Wild	Fruit	Decoction; taken orally for helping digestion, treating for diarrhea, dysentery, stomachache, dyspepsia, retention of food
Rosaceae	*Sanguisorba officinalis* L.	Diyu地榆	*	Herb	Wild	Root	Decoction; taken orally for clearing heat and detoxicating, cooling blood and hemostasis, treating for hypertension, hematemesis, hemoptysis, metrorrhagia and metrostaxis, dysentery with bloody stool, hemorrhoids; Pounded fresh part applied on the affected area for sore and furuncle, fester, snakebite, burn
Rutaceae	*Zanthoxylum bungeanum* Maxim.	Huajiao花椒	***	Tree	Wild	Fruit, root, seed, leaf	Decoction; taken orally for preventing thrombosis, analgesia, anti-inflammatory effect, promoting appetite and digestion, strengthening spleen and stomach, treating for diarrhea, vomiting; Fumigating and washing the affected area for pruritus of vagina
Rutaceae	*Zanthoxylum simulans* Hance	Yehuajiao野花椒	*	Tree	Wild	Fruit, root, stem, leaf	Decoction; taken orally for detumescence and analgesia, regulating the flow of vital energy, removing cold and detoxicating, diuresis and promoting stomach, anti-inflammatory effect, treating for diarrhea and vomiting
Selaginellaceae	*Selaginella tamayiscina* (Beauv.) Spring	Juanbai卷柏	*	Herb	Wild	Whole plant	Decoction; taken orally for dysmenorrhea and amenorrhea, traumatic injury, charcoal of it treating for vomiting blood, Metrorrhagia
Smilacaceae	*Smilax riparia* A.DC.	Niuweicai牛尾菜	*	Herb	Wild	Root or rhizome	Decoction; taken orally for dispersing blood stasis, relieving rigidity of muscles and activating collaterals, antioxidation, anti-tumor effect, promoting blood circulation, dispelling wind, analgesia effect, treating for rheumatic arthritis, arthralgia and myalgia, lumbar muscle strain
Solanaceae	*Datura stramonium* L.	Mantuoluo曼陀罗	*	Herb	Wild	Flower, leaf and seed	Decoction; taken orally for sedative and analgesic effect, anesthetic and anticonvulsive effect, relieving asthma and cough, treating for epilepsy, asthma; Washing in soup for rheumatalgia
Solanaceae	*Lycium chinense* Miller	Gouqi枸杞	*	Shrub	Wild	Fruit, bark	Decoction; taken orally for moistening the lung and promoting kidney, improving eyesight and cooling blood, anti-aging and anti-tumor effect, anti-inflammatory effect, clearing heat and relieving a cough, preventing atherosclerosis, protecting liver and cardiovascular, treating for hypertension, hyperlipidemia and diabetes, liver cancer
Solanaceae	*Solanum nigrum* L.	Longkui龙葵	*	Herb	Wild	Whole plant	Decoction; taken orally for clearing heat and detoxicating, promoting blood circulation, detumescence, diuresis and resolving phlegm, relieving itching, anti-inflammatory and anti-tumor effect, treating for chronic bronchitis, hypertension, acute nephritis, bladder cancer, dysuria, dysentery, leukorrhea, urinary tract infection; Pounded fresh part applied on the affected area for urticaria, cervical erosion, eczema, venomous snake bite, furuncle
Ulmaceae	*Ulmus parvifolia* Jacq.	Langyu榔榆	*	Tree	Wild	Bark, leaf, stem	Decoction; taken orally for clearing heat and detoxicating, cooling blood and hemostasis, detoxification, relieving swelling, treating for heat strangury, difficult urination, back pain, dysentery; Pounded fresh part applied on the affected area for acute mastitis, burn, sore
Urticaceae	*Urtica fissa* E. Pritz.	Qianma荨麻	*	Herb	Wild	Whole plant	Decoction; taken orally for dispelling wind and eliminating dampness, arresting convulsion, diuresis and hemostasis, preventing hair loss, removing phlegm, treating for rheumatoid arthritis, febrile convulsion, dyspepsia; Pounded fresh part applied on the affected area for seborrheic dermatitis, urticaria, dandruff and snakebite
Violaceae	*Viola yedoensis* Makino.	Zihuadiding紫花地丁	*	Herb	Wild	Whole plant	Decoction; taken orally for clearing heat and detoxicating, cooling blood and detumescence, anti-inflammatory and antibacterial effect, antioxidation, treating for carbuncle abscess, furuncle, pyogenic infections, acute mastitis, periappendicular abscess, snakebite
Vitaceae	*Vitis amurensis* Rupr.	Shanputao山葡萄	*	Liana	Wild	Root, stem, fruit	Decoction; taken orally for clearing heat, diuresis, dispelling wind and relieving pain, treating for urinary tract infection, dampness-heat of bladder, abdominal pain, headache, traumatic pain, rheumatalgia

The life habits of medicinal plants in SDSTS are mostly herbaceous (70%), represented by 86 species. Other forms like trees, lianas and shrubs were represented with 15 species (12%), 10 species (8%) and 12 species (10%), respectively.

It is reported that the whole plant (referring to the aerial part of the plant in the present research) and other parts such as bark, root, leaf, fruit, seed, stem, bulb, flower, kernel, rhizome, thorn and tuber are collected as medicine. Even though about 13 different plants parts were reported to be used for remedy preparation in different ways, a larger proportion (30.8%) of the preparations were obtained from the whole plant followed by root (18.3%) and fruit (11.2%). The leaf was used for 8.3% preparations and stem for 6.5%, rhizome and seed part both were 5.9%, whereas bark, flower, tuber, bulb, thorn and kernel were 5.3%, 3%, 1.8%, 1.2%, 1.2% and 0.6%, respectively.

Among them, 97 species were reported to be used with only one part. Eleven species were used with two parts, and there were 7 species with three parts used, 5 and 2 with four and five parts, respectively. *Celastrus orbiculatus* Thunb. and *Morus alba* L. have the most parts used for medicinal purposes.

### Modes of preparation and administration

As herbalists reported in the study area, ways of preparing remedies include three main types: decoction, pounding and cooking. The major way of herbal medicine preparation was decoction through boiling with clean water including 123 species, from those, 39 species have another method that is crushing the plant parts. *Juglans regia* L. tends to be eaten directly or cooked with meat.

The majority of the reported plant species were commonly processed into decoctions, which have been reported as one of the best approaches to extracting beneficial secondary metabolites [35, 36]. Some species for both medicinal and food purposes belong to this category. Representatives include rhizomes of *Polygonatum*, and roots of *Platycodon grandiflorus* and *Arctium lappa*.

Results of analysis of route of administration of medicinal preparations revealed that oral application was the most common route of administration (121 preparations), combined with external application (39 preparations), fumigating (3 preparations) and washing in soup (9 preparations), which is derived from decoction preparation. They vary based on different collocations with each other. All species but *Incarvillea sinensis* Lam. were reported to be orally administered. Some of them have more than one route. *Artemisia argyi* Levl. et Van were also hung on doors as antibiosis and repellents.

### Major types of diseases occurred in the study area

**Fig. 3:**
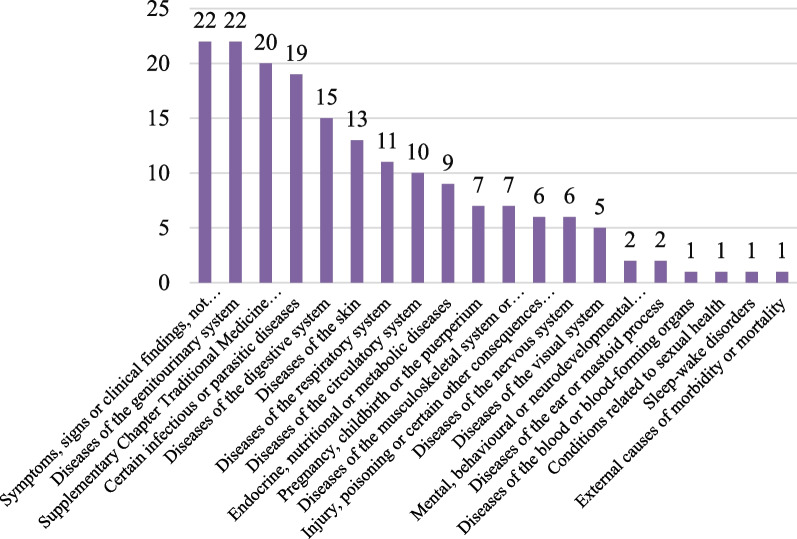
Classifications of diseases based on ICD-11

There were 180 mentioned human diseases, and the top 29 kinds of them are listed in Table 2. All of the diseases were identified on the International Classification of Diseases 11th Revision (ICD-11) System according to the description and explanation from herbalists, and classified into 20 kinds as shown in Fig. 3. The most cited health problems belong to “Symptoms, signs or clinical findings, not elsewhere classified” (22 diseases) and “Diseases of the genitourinary system” (22 diseases), “Supplementary Chapter Traditional Medicine Conditions-Module I” (20 diseases) and “Certain infectious or parasitic diseases” (19 diseases) Chapter.

**Table 2:** List of top 29 most treated human ailments in the study area

Clinical terms	ICD-11 chapter	ICD-11 code	Frequency
Dysentery	Certain infectious or parasitic diseases	1A40.Z	30
Hypertension	Diseases of the circulatory system	BA00	22
Hemorrhinia	Symptoms, signs or clinical findings, not elsewhere classified	MG27	21
Traumatic injury	Injury, poisoning or certain other consequences of external causes	ND56.Z	19
Swelling and pain in throat	Diseases of the respiratory system	CA02.Z	19
Sore and furuncle	Supplementary Chapter Traditional Medicine Conditions—Module I	SB4Z	19
Cough and asthma	Diseases of the respiratory system	CA23.3	17
Chronic bronchitis	Diseases of the respiratory system	CA20.1	16
Diarrhea	Symptoms, signs or clinical findings, not elsewhere classified	ME05.1	15
Dysuria	Diseases of the genitourinary system	MF50.6Z	14
Rheumatic arthritis	Diseases of the musculoskeletal system or connective tissue	FA20	14
Rheumatalgia	Symptoms, signs or clinical findings, not elsewhere classified	ME83	13
Cough due to excessive phlegm	Symptoms, signs or clinical findings, not elsewhere classified	MD10	12
Hematemesis	Symptoms, signs or clinical findings, not elsewhere classified	ME24.A5	11
Eczema	Diseases of the skin	EA8Z	11
Snakebite	External causes of morbidity or mortality	PA78	11
Diabetes	Endocrine, nutritional or metabolic diseases	5A14	10
Edema	Symptoms, signs or clinical findings, not elsewhere classified	MG29.Z	9
Constipation	Symptoms, signs or clinical findings, not elsewhere classified	ME05.0	9
Insomnia	Sleep–wake disorders	7A0Z	9
Dermatitis	Diseases of the skin	EA8Z	9
Pyogenic infections	Certain infectious or parasitic diseases	1C44	9
Jaundice	Symptoms, signs or clinical findings, not elsewhere classified	ME10.1	8
Arthralgia and myalgia	Symptoms, signs or clinical findings, not elsewhere classified	ME82	8
Toothache	Diseases of the digestive system	DA0A.Y	8
Malaria	Certain infectious or parasitic diseases	1F4Z	8
Acute mastitis	Diseases of the genitourinary system	GB21.Z	8
Irregular menstruation	Diseases of the genitourinary system	GA20.3	8
Wind-heat common cold	Supplementary Chapter Traditional Medicine Conditions—Module I	SF8A	8

### Edible use of medicinal plants

All 123 medicinal plant species recorded for treating human ailments in the study area were also cited for the edible use. The medicinal and edible plant species, as well as edible methods for them, are summarized in Table 3.

**Table 3:** List of plants for both medicinal and edible purposes

Scientific name	Folk Chinese name	Edible purposes		Habit	Medicinal purposes	
		Parts used	Preparations		Parts used	Preparations
*Abutilon theophrasti* Medicus	qing ma zi	Fruit, tender leaf	Tender leaf: juiced, tea substitute Fruit: eaten raw	Herb	Seed	Decoction
*Achyranthes bidentata* Blume	shan xian cai	Tender leaf	Stir-fried	Herb	Root	Decoction
*Albizia julibrissin* Durazz	ye he shu	Tender leaf, flower	Tea substitute, stewed as tonic soup	Tree	Flower, bark	Decoction Pounded fresh part applied on the affected area
*Allium macrostemon* Bunge	shan suan	Tender leaf, bulb	Stir-fried, dipped in sauce, used for seasoning, pickled	Herb	Bulb	Decoction
*Arctium lappa* L		Tender stem, tender leaf and root	Tender leaf and stem: juiced, stir-fried Root: stewed as tonic soup, tea substitute	Herb	Fruit, root, leaf, flower	Decoction Pounded fresh part applied on the affected area
*Bassia scoparia* (L.) A.J.Scott	tie sao zhou	Tender stem, tender leaf and seed	Tender stem and leaf: blanched before made into salad, stir-fried Seed: oil manufacture	herb	Whole plant	Decoction Pounded fresh part applied on the affected area
*Cirsium arvense* var. *integrifolium* C. Wimm. et Grabowski	ci ji cai	Tender stem, tender leaf	Tender stem and leaf: blanched before made into salad, stewed as tonic soup	Herb	Leaf, root	Decoction
*Cuscuta chinensis* Lam.	tu er si	Tender leaf	Tea substitute	Herb	Seed	Decoction Pounded fresh part applied on the affected area
*Diospyros kaki* Thunb.		Fruit, leaf	Fruit: eaten raw Leaf: tea substitute	Tree	Root, fruit, leaf	Decoction
*Forsythia suspensa* (Thunb.) Vahl.	Leaf	Stewed as tonic soup, tea substitute	Shrub	Fruit	Decoction	
*Hemerocallis fulva* (L.) L.	shan jin zhen	Flower	Stir-fried, stewed as tonic soup, blanched before made into salad	Herb	Whole plant	Decoction
*Juglans regia* L.	he tao mao mao	Fruit, flower	Fruit: eaten raw, oil manufacture, juiced, boiled and consumed as salad, dried and made into nuts Flower: stir-fried, made into salad after soaked in water for a few days	Tree	Kernel	Taken as nut or cooked with meat
*Leonurus japonicus* Houttuyn	rao wei, you bin chou cao	Tender leaf	Blanched before made into salad, stewed as tonic soup, Stir-fried with eggs, tea substitute	Herb	Whole plant	Decoction
*Lilum browaii* var. *viridulum* Baker	Bulb, flower	Bulb: stir-fried, stewed as tonic soup, tea substitute Flower: tea substitute	Herb	Bulb	Decoction	
*Lonicera japonica* Thunb		Flower	Tea substitute, stewed as tonic soup	Liana	Flower, stem, fruit	Decoction
*Morus alba* L		Fruit, bark and tender leaf	Fruit: eaten raw Bark: tea substitute Tender leaf: tea substitute, blanched before made into salad, stewed as tonic soup	Tree	Leaf, root, stem, fruit, bark	Decoction Pounded fresh part applied on the affected area
*Periploca sepium* Bunge	yang tao, wu bei zi	Tender leaf	Boiled and consumed as salad	Shrub	Bark	Decoction
*Pistacia chinensis* Bunge	huang lian	Tender leaf, seed	Tender leaf: tea substitute, stir-fried, pickled Seed: oil manufacture	Tree	Bark, leaf	Decoction
*Plantago asiatica* L	niu lun cai, yang ti miao, zhu er duo	Tender leaf, seed	Tender leaf: blanched before made into salad, stewed as tonic soup, stir-fried Seed: tea substitute	Herb	Whole plant	Decoction Pounded fresh part applied on the affected area Washing in soup
*Platycodon grandiflorus* (Jacq.) A. DC	Tender leaf, root	Tender leaf: stir-fried Root: pickled	Herb	Root	Decoction	
*Polygala sibirica* L		Leaf, root	Leaf: stir-fried Root: stewed as tonic soup, liquor brewing	Herb	Root	Decoction
*Polygonatum odoratum* (Mill.) Druce	Leaf, root	Leaf: stir-fried Root: tea substitute, stewed as tonic soup	Herb	Rhizome	Decoction	
*Polygonatum sibiricum* Delar. ex Redoute	ye sheng jiang	Tender leaf, root	Tender leaf: stir-fried, tea substitute Root: boiled as congee	Herb	Rhizome	Decoction
*Rumex crispus* L	(niu) she tou cai	Leaf, root	Blanched before made into salad, stir-fried, stewed as tonic soup, used for seasoning	Herb	Root	Decoction Pounded fresh part applied on the affected area
*Taraxacum mongolicum* Hand.-Mazz	bo bo ding cai	Tender leaf, whole plant	Tender leaf: tea substitute Whole plant: blanched before made into salad, stewed as tonic soup, dipped in sauce, stir-fried	Herb	Whole plant	Decoction Pounded fresh part applied on the affected area
*Takhtajaniantha austriaca* (Willd.) Zaika, Sukhor. & N. Kilian	lu lu cao	Stem, leaf and root	Eaten raw and dipped in sauce, stir-fried	Herb	Whole plant	Decoction
*Vicia amoena* Fisch	Fruit	Boiled	Herb	Whole plant	Decoction Fumigating and washing the affected area Pounded fresh part applied on the affected area	

### The most important medicinal plants in SDSTS

*Bupleurum scorzonerifolium* Willd., *Bupleurum chinense* DC., *Forsythia suspensa* (Thunb.) Vahl, *Zanthoxylum bungeanum* Maxim. and *Periploca sepium* Bunge were the highly utilized and the most significant medicinal species in SDSTS. As native species, they indicate that the local flora of SDSTS delivers significant medicinal uses. There even have Products of Geographical Indication of them—“Shexian Chaihu”, “Shexian Lianqiao” and “Shexian Huajiao,” in which *Chaihu* refers to the dried roots of *Bupleurum*, *Lianqiao* to the dried fruits of *Forsythia* and *Huajiao* to *Zanthoxylum* fruits, respectively. *Juglans* and *Taraxacum* have a great symbiosis highly acclimatized to the local mountainous environment [37]. For all native species above, the local has a large area under cultivation and a long history of planting, as well as rich experience in cultivation and production other than in the wild. For example, there was a cultivated area of 66.7 square kilometers for *Bupleurum* and 80 square kilometers for *Forsythia* in Shexian County by 2020, attaining a total production value of up to 247 million RMB (36.9 million USD). Particularly, *Bupleurum* and *Forsythia* are prominent varieties of the characteristic industry for traditional Chinese medicine, promoting the implementation of strategy for rural revitalization in Shexian County. They not only contributed to the development of the industry of traditional Chinese medicinal materials in Shexian County, but also promoted the agricultural production and farmers' income. The locals depend on them for living.

**Fig. 4:**
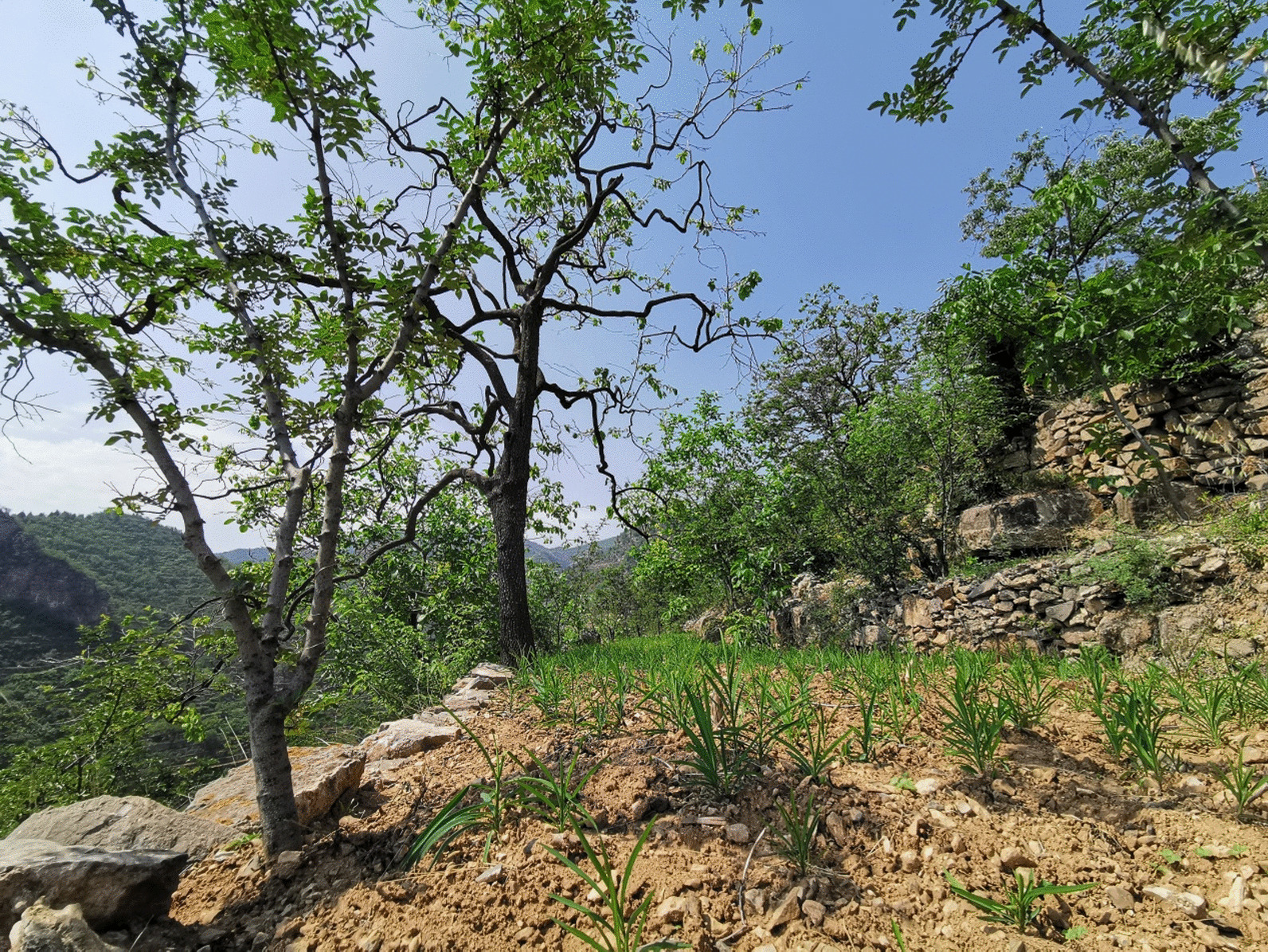
*Bupleurum* seedlings were cultivated in SDSTS in spring, forming an agroforestry system with *Zanthoxylum bungeanum* and *Diospyros kaki*

*Bupleurum* exists on the slopes and in the shrubs at an elevation of 400 to 1500 m above sea level in the wild. They were mainly cultivated in Piancheng and Guanfang townships. With a yield of 1600 kg per square kilometer, the dried roots of *Bupleurum* were sold to domestic traditional Chinese medicine markets and even overseas. Previous studies have also shown that the total flavonoids in different parts of *Bupleurum* in Shexian County had strong antioxidant activities and great potential as a natural antioxidant in food [38]. In SDSTS, some species have been cultivated traditionally (Fig. 4).

## Discussion

### A verification for local traditional Chinese medicine capacity

Shexian County has a sound foundation of traditional Chinese medicine whether in the history or in modern life [39], which are the basic materials for National Traditional Chinese Medicine with a large cultivated area, such as *Bupleurum chinense* DC., *Forsythia suspensa* (Thunb.) Vahl, *Zanthoxylum bungeanum* Maxim., *Belamcanda chinensis* (L.) Redouté, *Scutellaria baicalensis* Georgi, *Nepeta cataria* L., *Salvia miltiorrhiza* Bunge and *Anemarrhena asphodeloides* Bunge. In Shexian Dryland Stone Terraced System, a total of 123 medicinal plant species were reported to treat human ailments traditionally, indicating the presence of a considerable diversity of medicinal plants and a huge exploiting potential of traditional Chinese medicine there. At the same time, this study can provide the local traditional Chinese medicine industry a valuable reference data. The existence and utilization of a large number of medicinal plants might demonstrate that the majority of people continue to employ indigenous medicinal practices to date. Patients could collect medicinal herbs to treat themselves after the herbalists gave them diagnoses in a descriptive way when the ailments they got are common like the traumatic injury, fever, swelling and pain in throat, snakebites, cold and cough, and so on. The reality shows that local people have got a good command of the relevant knowledge and skills while using medicinal plants because they knew what kind of herbs can treat the ailment they got, where to collect and how to process them.

### A legendary history of “*Bupleurum* injection” in Shexian County

The wild *Bupleurum* has a time-honored history and civilization as the speciality in Shexian County. During the Anti-Japanese war period (1937–1945), the local wild *Bupleurum* resources were used to research and develop into “*Bupleurum* injection” by the Chinese Eighth Route Army’s 129 Division, the troops commanded by two famous leaders of the People’s Republic of China, Liu Bocheng and Deng Xiaoping. Liu was conferred on marshal in 1955, and Deng became the top Chinese leader in 1978. *Bupleurum* injection had been used to cure influenza and fever, and treat malaria, making a great contribution to the victory of the Anti-Japanese War. The adequate medicinal plants around the Taihang Mountains were widely applied by the Chinese Eighth Route Army to the Anti-Japanese war as crucial substitutes when the Japanese army cut off their supplies and the drugs such as sulfonamides, aspirins and quinines were severely scarce. In order to meet the challenges of war, like the storage and transportation, the wild *Bupleunum* resources had been developed into injections led by Gang Han in 1941, the director of LiHua Pharmaceutical Factory. That was how the first intramuscular injection of the traditional Chinese medicine was brought forth. As the first traditional Chinese medicine injection, it was a remarkable invention for the manufacture of traditional Chinese medicines using a western way, making it possible for the traditional Chinese medicine to give first-aid treatment. The manufacture of “*Bupleurum* injection” survived the Japanese army’s medicine blockade, supporting the Anti-Japanese war to a great extent. The valuable history of the wild *Bupleurum* is not only an important component of local cultural heritage but a typical example to demonstrate what an irreplaceable position of traditional medicinal plants played in the history of the world.

### The medicinal and edible plants

There are 28 species listed in Table 3 used for both medicine and food, which indicated that the roles indigenous people taking plant resources are sufficient and diverse. As one of three precious local specialties in Shexian County, *Zanthoxylum bungeanum* Maxim. possesses huge and various values including promoting appetite and digestion, strengthening spleen and stomach for medical function, being a natural flavor in daily life to make the dish taste better, and having insecticidal and antisepsis effect using in food and clothing. *Aquilegia viridiflora* Pall. is similar. Local people picked the wild for promoting blood circulation, treating mainly for gynopathy like abnormal menstruation. This species also served as palatable homemade dishes. The specific culinary procedures for it are as follows: firstly clean up the plants, then boil them with water, and finally put them into the millet congee, or cooked as the vegetable soup directly. As is often the case with *Hemerocallis citrina* Baroni and *Taraxacum mongolicum* Hand.-Mazz., they are common on the dining tables as salads with sauce, or as a kind of dumpling stuffing. In the long-term interaction of living and thriving, much traditional knowledge about wild medicinal plants and edible plants has been developed and accumulated by the local people owing to this northerly limestone mountainous area, poor transportation and abundant natural resources. As far as SDSTS is concerned, it has been undergoing drought and flood disasters and had the cruel wars. Therefore, the medicinal and edible uses of wild plants in local are likely to be the most primary use patterns. It is equally important and urgent to study edible plants in SDSTS and their traditional knowledge.

### Agrobiodiversity in SDSTS

According to Food and Agriculture Organization of the United Nations (FAO), agrobiodiversity is defined as “the variety and variability of animals, plants and micro-organisms that are used directly or indirectly for food and agriculture, including crops, livestock, forestry and fisheries. It comprises the diversity of genetic resources (varieties, breeds) and species used for food, fodder, fiber, fuel and pharmaceuticals. It also includes the diversity of non-harvested species that support production (soil micro-organisms, predators, pollinators), and those in the wider environment that support agro-ecosystems (agricultural, pastoral, forest and aquatic) as well as the diversity of the agro-ecosystems.” Agrobiodiversity is a vital sub-set of biodiversity, guaranteeing the livelihoods and food security of local communities and providing multiple ecological functions [40]. Agrobiodiversity is regarded as the central to overall biodiversity, but overlooked to a great extent and under threat [41]. Issues on agrobiodiversity in agricultural heritage sites are particularly highlighted.

The Globally Important Agricultural Heritage Systems (GIAHS) program of FAO aims to identify agricultural systems of global importance, preserve landscape, agrobiodiversity and traditional knowledge and apply the principles of dynamic conservation to promote sustainable development. Agrobiodiversity is one of the most important components of agricultural heritage systems. Many farmers who live in difficult environments, rely on diverse traditional varieties of crops. This helps them maintain their livelihood in the face of pathogen infestation, uncertain rainfall and fluctuation in the price of cash crops, sociopolitical disruption and the unpredictable availability of agro-chemicals. The need for different agricultural products at different times and agroecological conditions, however, is more clearly and commonly noted than the others of agrobiodiversity. As a result, there are now considerable studies about the amounts of crop genetic and species diversity maintained in agricultural heritage systems and the reasons for this [16, 17, 42, 43]. However, mostly wild species were neglected in those studies on agrobiodiversity. More accurately, for those that are frequently found next to the main staple or cash crops, and native plants emerged spontaneously in the system. They often appeared in daily life and their importance is often misjudged.

Fortunately, agrobiodiversity in SDSTS has been emphasized since it was assigned as a China-nationally Important Agricultural Heritage System site in 2014. The local people traditionally conserved their crop genetic resources in the stone terraced fields. Previous research conducted in Wangjingzhuang Community revealed that there is rich agrobiodiversity in SDSTS. Researchers identified 77 species in 57 genera and 26 families, including 171 landraces cultivated or managed in the Shexian Dryland Terrace System. These plant species covered 15 grain crops, 31 vegetables, 5 oil-bearing crops, 14 fruits, and 12 medicinal, textile, and tobacco plants. The landraces discovered in SDSTS are grains (62), vegetables (57), fruits (33), oil-bearing crops (7), and others (12 varieties of medicinal, textile, and tobacco plants) [17]. More importantly, the present study dealt with wild plants in SDSTS, focusing on their medicinal plants. To compare with other GIAHS sites, our research in SDSTS initiated an aspect of agrobiodiversity investigation, which refers to medicinal plants traditionally used by the local people. Our findings would probably provide a reference for other GIAHS sites.

## Conclusions

Shexian Dryland Stone Terraced System (SDSTS) retains substantial medicinal plants and associated traditional knowledge, which has been reflected in our surveys. There were 123 medicinal plant species belonging to 51 families documented from SDSTS. Asteraceae is the largest family with 16 species followed by Fabaceae, Lamiaceae and Ranunculaceae (8 species). The majority of the medicinal plants were commonly processed into decoctions. And 180 diseases affecting humans were reported to be treated traditionally with medicinal plants in SDSTS. It is the first case study to identify medicinal plants that were traditionally used in agricultural heritage sites.

Designated as GIAHS by FAO, Shexian Dryland Stone Terraced System has been proven to be of global importance. This study helps to fill a gap in surveys of wild plant resources in SDSTS. The ethnobotanical survey provides a foundation and reference for the conservation and sustainable development of agrobiodiversity in GIAHS.

## Data Availability

All data, materials, and information are collected from the study sites.
